# Waves in Extremities: A Rare Report of Isolated Isaacs' Syndrome

**DOI:** 10.7759/cureus.4687

**Published:** 2019-05-17

**Authors:** Syed Hamza Bin Waqar

**Affiliations:** 1 Internal Medicine, Civil Hospital Karachi, Dow University of Health Sciences, Karachi, PAK

**Keywords:** neuromuscular hyperexcitability, neuromyotonia, myokymia, cold-induced allodynia, anti-vgkc, caspr2, ísaacs' syndrome

## Abstract

Isaacs' syndrome is a rare neuromuscular hyperexcitable syndrome with myriad manifestations ranging from motor and sensory to autonomic presentations, leading to diagnostic challenges. Among the commonest forms, a tetrad of stiffness, myokymia (muscle twitching at rest), weakness, and psuedomyotonia (delayed muscle relaxation) is almost always present. Herein, we report a case of a 16-year-old male who presented to the neurology consult service with intense wave-like pain in the lower extremities with desquamating rash and cold-induced allodynia. Investigations were significant for raised CK levels, positive autoimmune panel, and anti-voltage-gated potassium channel (anti-VGKC) antibody that is involved in 35% reported cases of Isaacs' syndrome, with electrodiagnostic studies peculiar for Isaacs syndrome with negative imaging results. He was followed up on a long cocktail course of immunosuppressive, anticonvulsive medications, and immunoadsorption plasmapheresis (IAP) for 11 months with complete remission.

## Introduction

Neuromuscular hyperexcitability has been documented in many disorders. Isaacs' syndrome documented first in 1961 by Isaacs; is one of a kind. It is a humoral-mediated neuro-excitability syndrome, mostly affecting the myelinated peripheral nerve fibers blocking up the outward going potassium current [1]. Such blockage results in a condition called neuromyotonia, described as continuous and spontaneous muscle fiber activity, which is the major manifestation of Isaacs' syndrome. At times, it is also compounded by autonomic and central nervous disturbances creating a diagnostic dilemma for physicians. The main etiopathogenesis lies in the autoimmune process that can either be due to an inherited tendency or acquired as a result of paraneoplastic syndromes and at times remains isolated. The autoimmune phenomenon involves anti-voltage-gated potassium channel antibodies in most cases, which cause disruption at the neuromuscular level, making it somehow manageable with immunosuppressive therapies [1]. Herein, we report a case of a 16-year-old male who presented with severe pain and stiffness in extremities with wave-like sensation in the muscles. He was diagnosed with Isaacs’ syndrome and underwent remission on an intensive regimen of immunosuppressants.

## Case presentation

A previously healthy 16-year-old male with no known co-morbid presents to tertiary care hospital with intense pain, stiffness, and fasciculations in bilateral lower extremities for over a month. His pain started gradually and got progressively continuous in a matter of days. It was burning in nature, non-radiating, moderate in intensity which incremented as the patient walked over a smaller distance. It wasn’t relieved at rest or with any of the prescribed pain medications he took. He also had stiffness with the pain, more-so below knees that at thighs. Additionally, he complained of pain on touch and had a sensitivity to cold which aggravated his discomfort level. The discomfort was described as a wavy sensation in extremities. The patient also noted episodic twitching all over the body especially in the calf, chest and around the corners of his mouth for over a month, which worsened on movement. There was no urinary or fecal incontinence, fits or speech problems, blurry vision or visual acuity changes. He had no significant past medical, surgical, or family history of any known illness. Sleep was disturbed because of discomfort and pain in calf muscles and nocturia.

On admission, he was restless and in significant discomfort but alert, and oriented with no mood alterations. The patient was afebrile with a pulse of 114 beats per minute (BPM), BP of 155/100 mmHg, and respiratory rate of 20/minute. Power was normal in upper extremities and lower extremities above the knee but four out of five below knee bilaterally. Reflexes were active in grade in brachioradialis, biceps, triceps, and ankle but diminished at the knee. Plantar reflex was bilaterally mute. The bulk was bilaterally symmetrical and tone, normal on assessment. He also reported decreased pin-prick sensation in toes and fingers bilaterally in both lower extremities but had intact joint position sense. Cerebellar signs of co-ordination and cranial nerves two till twelve were intact with no observed nystagmus or tremors. The patient had an antalgic gait. Desquamating, blanchable erythema in both feet was noted. There were no signs of jaundice, edema, visceromegaly, or lymphadenopathy. Abdominal, respiratory, and cardiovascular were normal on physical examination.

The complete blood count (CBC) showed total leukocyte count (TLC) of 4.36 x 10^9^/L, with differential showing neutrophil percentage being 85.6% and lymphocyte being 10%, platelet count of 447 x 10^9^/L, an erythrocyte sedimentation rate (ESR) of 12 and C-reactive protein (CRP) of 0.7. Prothrombin time (PT) and activated partial thromboplastin time (APTT) were 9.7 and 20.2 seconds, respectively. The glycosylated hemoglobin (HbA1c) was 4.8%. Assay for anti-neuronal antibodies that is anti-voltage-gated potassium antibodies was sent and turned out to be positive. Immunology panel including anti-rheumatoid factor, anti-smooth muscle antibodies (ASMA), anti-mitochondrial antibodies (AMA), anti-liver kidney microsomal antibodies, anti-cyclic citrullinated peptides (anti-CCP), anti-nuclear antibody (ANA), anti-cytoplasmic antibodies (ANCA), anti-Ro, anti- La, anti-acetylcholine receptor antibody (anti-AchR), anti-double-stranded DNA antibody (anti-dsDNA), and complement and cryoglobulin levels turned out to be negative. Electrolytes, liver function, and thyroid function test were normal. Serologic tests for Hepatitis B surface antigen (HBsAg), and Hepatitis C antibody (anti-HCV) were insignificant. Serum creatine phosphokinase (CPK) levels and aldolase levels were marginally elevated.

Concentric single fiber electromyography (EMG) was performed, which showed neuromyotonic discharges that were irregular and continuous with triplets and multiplets having high intra-burst frequencies of 30 to 250 Hertz (Hz) interposed between normal motor unit potentials (MUPs) of electrical silence and reduced recruitment. Nerve conduction study (NCS) test was done, which was significant for showing mild neuropathy. 

Computed tomography (CT) chest and CT abdomen/pelvis, ultrasound (US) abdomen, magnetic resonance imaging (MRI) of the brain and spine were done and were negative for any tumoral growth.

He was followed initially on a cocktail regimen of phenytoin, gabapentin, tramadol, and prednisolone. As the discomfort level was significant, the patient was also put on sessions of immunoadsorption plasmapheresis, first consecutively for six weeks and then intermittently with the maintenance of phenytoin with remission of disease in eleven month period. Blood pressure elevations were constantly managed in the background and appropriate investigations involving plasma metanephrines, cortisol levels, aldosterone and renin levels, Echocardiography, and ultrasound renal artery Doppler was done but turned out to be insignificant suggesting autonomic disturbance.

## Discussion

Peripheral nerve hyperexcitability (PNH) is a known phenomenon that is described as a shift of the resting membrane potential to lesser negative values resulting in the exponential firing of neurons in the peripheral nervous system. One such condition is associated with Isaacs’ syndrome which is a humoral-mediated dendrotoxin-sensitive fast potassium channelopathy. Antibodies against voltage-gated potassium channel (anti-VGKC) have been reported in 35% of such cases making it one of the main causes of Isaacs’ pathogenesis as in this case [2]. Such antibodies are known to reduce the density of functional VGKCs, resulting in a greater expression of sodium channels and reduction in outflux of potassium rendering neuron sensitive to neuro-excitability and ectopic discharges. Research has shown that other antibodies causing Isaacs’ are directed against protein complexed to potassium channel rather than the whole channel by itself. These anti-neuronal antibodies are directed against leucine-rich glioma inactivated protein-1 (LGI1), contactin-associated protein-2 (Caspr2), and other unknown proteins that form a complex with VGKC [3].

The juxtaparanodal, pre and post-synaptic segments of myelinated fibers, especially the distal portion of the motor nerve, and/or the terminal arborization are typically involved in the origin of nerve excitability called as neuromyotonia, the primary manifestation of Isaacs’ syndrome, which is described as peripheral nerve-mediated spontaneous and continuous muscle fiber activity. This leads to a tetrad of muscle stiffness, myokymia (muscle twitching at rest), weakness and psuedomyotonia (delayed muscle relaxation) [4]. Electromyographic features of the spontaneous, continuous, irregularly occurring doublet or multiplet single motor unit (or partial motor unit) discharges, firing at a high intraburst frequency of 30 to 300 Hz, are peculiar for neuromyotonia [5].

In addition to peripheral involvement of the nervous system, Isaacs’ syndrome can also be associated with autonomic nervous system (ANS) resulting in symptoms like hyperhidrosis, skin changes, urinary incontinence, cardiac arrhythmia, and constipation and central nervous system (CNS) symptoms such as severe insomnia, hallucinations, impairment of short-term memory, and epilepsy. When involving the latter two systems, Isaacs’ syndrome is called Morvan syndrome. Mostly, anti-Caspr2 is involved with the central and peripheral nervous system with a slightly elevated risk of underlying malignancy while LGI1 is involved in limbic encephalitis [3,6].

At times, bulbar involvement can lead to respiratory compromise and pharyngeal insufficiency predisposing to aspiration [7].

Isaacs’ syndrome can either be inherited as an autosomal dominant trait with neuropathies, acquired as a paraneoplastic syndrome or can be idiopathic as in this case. Axonal neuropathies, in particular, can predispose paranodal regions to an autoimmune insult against VGKC. It has been noted that sensory symptoms develop earlier as compared to motor findings [8]. Certain autoimmune diseases as systemic lupus erythematosus, systemic sclerosis, dermatomyositis, Addison’s disease, Hashimoto's disease, and myasthenia gravis can also predispose to Isaacs' and have been reported in the literature [9].

For diagnosis, complete immunoassay of the autoimmune panel and infectious serologies is required with EMG and NCS to show typical electrodiagnostic features as discussed above. Imaging studies including CT scan chest, abdomen, and pelvis, ultrasound abdomen and magnetic resonance imaging of brain and spine should also be sought in all cases to exclude underlying malignancy. Tumor neo-antigens and tumor-associated antigens can predispose to autoimmune process resulting in Isaacs’ syndrome. Thymoma, Hodgkin lymphoma, plasmacytoma, hemangioblastoma, lung cancer, and lymphoblastic lymphoma have all been linked to Isaacs’ syndrome [4].

For treatment, immunomodulating therapy preferably plasmapheresis is considered as first-line therapy for most cases especially those who have autoantibodies against VGKC. Intravenous immunoglobulin (IVIG) has been used and has shown its effectiveness more so in cases where there were no reported anti-VGKC antibodies. Most of the time, phenytoin or carbamazepine is also added as maintenance which stabilizes the inactive form of sodium channels resulting in a voltage and frequency dependent reduction in trains of the action potential at higher frequencies. Other immunosuppressive like steroids or azathioprine can also be added depending on the treatment response. Most cases show a response in 13 months after which drugs can be withdrawn [1,10].

## Conclusions

Isaacs' syndrome can present with a multitude of possible combinations of symptoms leading to the diagnostic challenge. Despite being recognized by physicians, the complexity of presentation leads to inappropriate diagnosis. As mentioned, autoimmune etiopathogenesis in most cases can be countered by immunomodulating and immunosuppressive therapies making it a treatable condition. Underlying possible malignancies and autoimmune conditions should also be sought before marking a treatment plan. Further studies for Isaacs' syndrome should be conducted for a better understanding of etiology and for devising a proper treatment algorithm.
